# Consequences of nongenomic actions of estradiol on pathogenic genital tract response

**DOI:** 10.1186/1750-2187-8-1

**Published:** 2013-01-26

**Authors:** Paula Solar, Luis Velasquez

**Affiliations:** 1 Center for Integrative Medicine and Innovative Sciences, Facultad de Medicina, Universidad Andrés Bello, Echaurren 183, Santiago, Chile; 2 Centro para el desarrollo de la nanociencia y la nanotecnología (CEDENNA), Santiago, Chile

**Keywords:** Estradiol, Nongenomic, Genital tract, Pathogenesis, Immune response

## Abstract

Estradiol is a steroid hormone that regulates the structure and function of the female reproductive system. In addition to its genomic effects, which are mediated by activated nuclear receptors, estradiol elicits a variety of rapid signaling events independently of transcriptional or genomic regulation. These nongenomic actions influence the milieu of the genital tract, which changes the ability of pathogens to infect the genital tract. This review discusses our current knowledge regarding the mechanisms and relevance of nongenomic estradiol signaling in the genital tract that could change the ability of pathogens to invade epithelial cells. PubMed was searched through January 1980 for papers related to estradiol actions in the ovary, fallopian tube, uterus and cervix. The mechanisms conveying these rapid effects consist of a multitude of signaling molecules and include cross-talk with slower transcriptional actions. The nongenomic actions of estradiol that influence the infectious abilities of pathogens occur either directly on the genital tract cells or indirectly by modulating the local and systemic immune systems. Additional in-depth characterization of the response is required before the normal and pathological reproductive functions of the nongenomic estradiol pathway can be targeted for pharmacological intervention.

## Review

Despite the existence of public health programs and appropriate antibiotics for nearly 60 years, sexually transmitted infections (STIs) continue to be a problem worldwide [1]. In women, cervical infections may ascend into the upper genital tract to cause pelvic inflammatory disease (PID). PID encompasses a wide range of inflammatory conditions that can have potentially devastating reproductive consequences and that are recognized as important public health problems worldwide [2]. The immune system associated with the female genital tract (FRT) is particularly important in the development of this process because it is the first site of immunological contact with pathogens that cause STIs. Innate and early-induced immune responses may prevent the establishment of infection or reduce pathogenic replication until antigen-specific cells are recruited to the local site. The vagina, cervix, uterus and fallopian tubes contain the full complement of immune cells that confer both innate and specific immunity. However, the female reproductive tract is immunologically unique because it must tolerate allogeneic sperm and, in the upper tract, the conceptus. The numbers and activity of most of these cell types in the genital tract vary significantly throughout the menstrual cycle phases, and this variation is thought to be controlled by fluctuating levels of the female sex hormones estradiol and progesterone. For example, the expression levels of immunomodulatory genes change during the implantation window in the human endometrium [3]. Nuclear receptors such as ER-α and ER-β are DNA-binding proteins that recognize specific cis-acting hormone response elements upon ligand binding. They are typically located in the promoter regions of target genes. Thus, the most important feature of nuclear receptors is the ability to interact with DNA in response to hormone binding or other signals and to subsequently recruit multiprotein complexes that control gene expression [4]. However, not all of estradiol’s effects can be explained by the classical model of steroid action. Like every other steroid hormone, estradiol exerts rapid effects on diverse signaling pathways and second-messenger systems independently of transcriptional or genomic regulation [5-10]. These rapid responses are referred to as ‘non-classic,’ ‘nongenomic’ or ‘extranuclear’ steroid effects. Several criteria have been proposed that may facilitate the differentiation of nongenomic and genomic steroid actions. In general, nongenomic effects are (i) too rapid to be compatible with transcriptional activation and protein synthesis; (ii) not abolished upon the addition of transcriptional or translational inhibitors; (iii) sometimes observed in isolated cell membranes or in cells devoid of nuclei, such as erythrocytes and platelets; (iv) inducible by cell-impermeable steroid–protein conjugates; and (v) generally not inhibited by antagonists of nuclear steroid receptors [11].

In the literature, we found nongenomic effects of the estradiol in different target tissues, which can be related with the physiology of the female reproductive tract and with effects in the immune system. These actions are summarized in the Additional file 1.

On the other hand, we found effects of estradiol in the different sections of the female reproductive tract and in the immune system. These effects are summarized in the following subindexes by highlighting in the nongenomic effects at the end of each subscript. Finally, we relate these effects with the rapid effects in the immune system that can influence the pathogenic infection.

## Estradiol nongenomic actions on genital tract cells relevant to the pathogenic infection

The female reproductive tract environment can be subdivided into two major areas: the non-sterile vagina and ectocervix and the sterile endometrium and fallopian tubes. Each of these sections of the female reproductive tract is summarized in the subsections 1.1, 1.2 and 1.3.

### 1.1 Effects on vaginal and ectocervix cells

The vagina is the entrance to the female genital tract, and the vaginal portion of the ectocervix is structurally and immunologically similar to the vagina. The vagina’s luminal surface is lined with non-keratinized, squamous epithelium that is 150–200 μm thick, and the epithelial cells produce a hydrophilic layer of glycoprotein called the glycocalyx, which provides moisture and protection. Epithelial cell proliferation and maturation are under hormonal regulation, and at peak estrogen levels, the vaginal epithelium attains its maximum thickness with the superficial cells containing intracytoplasmic glycogen [12].

Infections in these sections are primarily restricted to *Candida albicans* and *Trichomonas vaginalis* in the vagina and Human Papilloma Virus (HPV) in the ectocervix. Even after stimulation, vaginal and ectocervical cells are negative in CD14 and TLR4 tool-like receptors (TLR) [13].

### 1.2 Effects on the endocervix

The endocervix is lined with simple epithelium composed of columnar cells with basally located nuclei and a fine granular cytoplasm filled with mucus droplets. These cells are interspersed with occasional ciliated, non-secretory columnar cells [14].

The latter cells mobilize and distribute the mucus. They may impede the ascent of bacteria because of their sweeping motion. The major pathogens that establish infections at this site are *Chlamydia trachomatis* and *Neisseria gonorrhoeae*.

Approximately 20–60 mg of cervical mucus is produced each day; the mucus provides a protective covering for the cervix and vagina and acts as a barrier to inhibit sperm and pathogens from entering the uterus [15]. The mucus changes from a viscous material to a watery, alkaline profuse fluid immediately prior to ovulation or under the influence of estradiol, facilitating sperm penetration [15]. The documented cervical mucus components include water (90–98%), low-molecular-weight components including organic compounds and inorganic ions and high-molecular-weight components including plasma proteins, secretory immunoglobulin, enzymes and bactericidal and bacteriostatic molecules as lysozyme, lactoferrin, zinc and the defensins human intestinal defensin-5 (HD-5) Human Beta defensin-1 and (HBD-1) [16,17]. Through a nongenomic pathway, estrogen affects ion concentrations in different cellular types. This in turn affects the amount of water through osmotic effects.

There are differences between the immune system components of epithelial, endocervical, ectocervical and vaginal cells. In the case of toll-like receptors, studies have reported a similar pattern of expression on endocervical epithelial cells and in the lower tract [18]. In contrast, the constitutive and induced cytokine profile in immortalized endocervical epithelial cells is substantially higher than in matched immortalized ectocervical and vaginal epithelial cells [19]. Furthermore, IL-6, IL-7 and RANTES were only synthesized by the endocervical line [19]. Beta-estradiol levels are significantly correlated with dendritic cell numbers, CD80 expression, IL-6 levels and IFN-gamma levels in women with fertility disorders [20].

### 1.3 Effects on the endometrium and fallopian tube cells

Endometrial epithelial cells synthesize an array of chemokines and cytokines, which play a central role in the controlled endometrial inflammatory response observed in implantation, pregnancy and menstruation. Estradiol regulates this phenomenon by modifying the expression and secretion of molecules that ensure fertilization and embryo viability. In the fallopian tube, estradiol likely regulates oviductal transport [21] by affecting muscle activity and ciliated cells [22-24], which is a nongenomic effect of estradiol. The nongenomic estradiol signaling is via cAMP signaling pathway [25] and the successive activation of cAMP-protein kinase A stimulate the signaling cascade by phospholipase C-IP3 and finally the release of intracellular calcium [26].

## Estradiol nongenomic action on the innate and humoral immune systems

### Effects on the innate system

Innate immune responses are activated by the binding of microbial pathogen-associated molecular patterns (PAMPs) to TLRs found on phagocytic and epithelial cells. This PAMP-TLR binding induces the secretion of antimicrobial peptides, cytokines and chemokines that attract phagocytic cells to the affected tissue, activating natural killer (NK) cells. Another mechanism of cytokine release to the extracellular medium is mediated by the inflammasome, which is a macromolecular structure that regulates the expression and release of IL-1β [27] and simultaneously stimulates the secretion of cyclooxygenase, TNF-ɑ and IL-8, causing inflammation and finally apoptosis. Estradiol affects both pathways. Changes in estradiol change the levels of TLR in the genital tract, which alters the response to invading pathogens. Expression variations have been recorded for TLR2, TLR3, TLR4 and TLR9; the levels increase during the perimenstrual period and decrease during the periovulatory period in the human endometrium [28].

Several natural antimicrobial peptides are expressed in the genital tract, and their expression levels are regulated in some cases by cyclic changes in estradiol and progesterone. For example, (HD-5) expression was highest during the early secretory phase of the cycle in endometrial cells. The concentrations of the secreted HD-5 peptide in cervicovaginal lavage were also highest during the secretory phase of the menstrual cycle. Variations of HD-2 have also been observed in which estradiol increases HD-2 secretion in uterine epithelial cells [29].

However, cytokine modifications that occurred in an estradiol-dependent manner and that were not blocked by antagonists of nuclear steroid receptors were recorded [30,31]. For example, TNF-ɑ, which is induced by the BCL2 apoptosis pathway, is suppressed in uterine epithelial cells [32]. One mechanism by which estradiol may generate this effect is by the MAPK phosphorylation cascade [33]. In contrast, the expression of IL-1β is increased by LPS in uterine epithelial cells [34]. A clear example of induction by cell-impermeable steroid–protein conjugates is the enhanced cytokine production that has been observed in splenic macrophages in male Sprague–Dawley rats [31].

The decreased transcriptional activity of the NF-kb signaling pathway is another previously reported estradiol effect [35]. Though this modification could alter the inflammatory response via a genomic pathway, the decreased activity of this transcription factor is mediated by the nongenomic pathway of estradiol.

**Figure 1:**
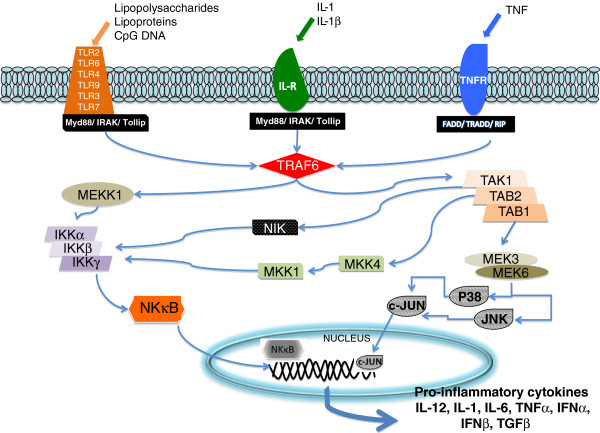
**Signal transduction pathway through TLR.** The signal transduction pathway was determined in human tissue in monocytes/macrophages, dendritic cells, intestinal epithelial and endothelial cells.

Estradiol has been reported to directly affect TRAF6 activity through a nongenomic pathway [36]. As observed in Figure 1, TRAF6 is a key factor for NF-kB activity and for the expression of pro-inflammatory cytokines.

Estradiol has general anti-inflammatory activity, which explains its effect on TRAF6, its effect on TLR expression and its effect on inflammasome activity. Estradiol inhibits the production of IL-1 mediated by the inflammasome, therefore affecting the caspase 1 pathway.

Estradiol also affects the innate immune system by altering the activity levels and numbers of NK cells in the FRT, which both increase during the mid-late secretory phase of the menstrual cycle. NK cells in the FTR are abundant in early pregnancy but progressively disappear from mid-gestation onwards and are absent at term. However, this effect is thought to be regulated by serpin peptidase inhibitor (PI-9) because PI-9 blocks the cell death induced by immune cells via the estrogen genomic pathway [37]. This fact is very important because estradiol could have effects in the fertility and immune systems by modifying levels of PI-9 and TRAF6.

### Effects on the humoral system

The regulation of antigen presentation is an important control point for the induction or suppression of adaptive immune responses. In the FRT, sex hormones influence antigen presentation by professional antigen-presenting cells (APCs: DC and macrophages) and non-conventional APCs found in the genital tract. Examples of this are estradiol-inhibited APCs in the vagina and the proliferation of B and T cells [38].

## Conclusions

The cycle stage or the presence of exogenous estrogen affects susceptibility to a number of STIs in humans and animal models [39]. Oral contraceptives increase the incidence of vaginal candidiasis [40], and the use of Norplant® increases the attachment and the invasiveness of *Neisseria gonorrhoeae*[41]. Furthermore, mice are the most susceptible to infection by *Neisseria gonorrhoeae* when their endogenous estrogen levels are the highest (during proestrus) [42]. In a mouse model, susceptibility to genital tract infection by *Mycoplasma hominis* and *Ureaplasma urealyticum* was affected following estradiol treatment [43]. In animal models, the grade of protection that is induced by vaccine protocols is influenced by the cycle stage [44].

The effect of estradiol in pathogenic infections is conditioned by its effects on the nongenomic pathway of the immune system and by its effect on cells of the reproductive tract. Estradiol modification changes ion channel activity by affecting the flow of vaginal secretions and by strongly influencing the intracellular concentration of Ca^++^. We now know that estradiol affects cAMP, inositol triphosphate, G protein and the MAPK pathway, which are all components of the calcium signaling pathway. The effect of estradiol in these molecules explains their impact on muscle contraction in the genital tract. The modification of these factors directly affects the infectivity of pathogens. Infectivity is also affected by estradiol because of its effect on the innate immune system.

**Figure 2:**
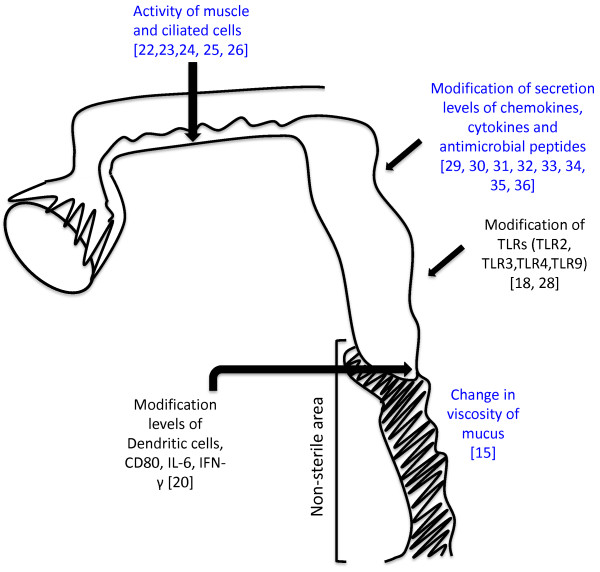
**Scheme of estradiol nongenomic effects in the female reproductive tract.** The scheme shows different sections of the female reproductive tract. Vagina and ectocervix correspond to non-the sterile area and endocervix, utero, fallopian tubes and ovary correspond to the sterile area. The scheme shows the effects produced only by the nongemonic pathway of estrogen in blue and the effects attributed to estradiol that are dependent of transductional pathways in black.

However, estradiol registered an effect in a large amount of cytokines and chemokines. Estradiol can modify the secretion of cytokines and chemokines by affecting NF-κB and TRAF6 and by modifying TLRs. The effects of estradiol are summarized in Figure 2. In this figure, we can see the effects of estradiol in different sections of the female reproductive tract. Note that only in the sterile area has estradiol affected the immunological system. This may be due to the absence of commensal flora, allowing it to modify the immune system.

## Abbreviations

STIs: Sexually transmitted infections; PID: Pelvic Inflammatory Disease; FRT: Female Genital Tract; Ca^++^: Calcium Ion; HPV: Human Papilloma Virus; TLR: Tool-like receptors; PAMPs: Pathogen-associated molecular pattern; NK: Natural Killer Cells; HD: Human Defensin.

## Competing interests

The authors declare that they have no competing interests.

## Authors’ contributions

PS drafted the manuscript, participated in the design of the study and participated in the analysis of the literature and signal transduction pathways. LV originated the study, participated in its design and coordination and helped draft the manuscript. All authors read and approved the final manuscript.

## Supplementary Material

Additional file 1 **Rapid effects of estradiol in target tissues.** Additional File 1 shows different rapid effects of estradiol reported in the literature. The file identifies the physiological effects, the related signaling pathway and the criteria of inclusion in the nongenomic action of estradiol. Click here for file
