# Supplementary material for: Consequences of nongenomic actions of estradiol on pathogenic genital tract response
Source: J Mol Signal. 2013 Jan 26;8:1. doi: 10.1186/1750-2187-8-1 (PMC3570385; doi:10.1186/1750-2187-8-1)
Supplement: Additional file 1 — Rapid effects of estradiol in target tissues. Additional File 1 shows different rapid effects of estradiol reported in the literature. The file identifies the physiological effects, the related signaling pathway and the criteria of inclusion in the nongenomic action of estradiol. [file 1750-2187-8-1-S1.pdf]

Additional file 1. Rapid effects of estradiol in target tissues.

Physiological action Cell	Tissue/ organism	Signaling pathway	Reference	Criteria of non genomic action
Unregistered	Pig granulosa cells	Mobilization of ion calcium Ca2+ triggered by inositol trisphosphate	Morley et al., 1992	ii
17 beta-estradiol was found to reduce Ba2+ entry reversibly via Ca2+ channels in acutely dissociated	Neostriatal neurons	G-protein activation	Mermelstein et al., 1996	i, iv
Increase in extracellular-regulated kinase (**ERK**) activity within 10 min	Chinese hamster ovary (**CHO**) cells	Phosphoinositide cascade and adenylate cyclase	Razandi et al., 1999	i
Nongenomic action of estrogen	Human SKBR3 breast cancer cells	Mediated by a G protein-coupled receptor Phosphorylation of ERK-1/-2	Filardo et al., 2000	i, ii, v
Suppression of tumor necrosis factor alpha-induced apoptosis	MCF-7 cells	Increase in Bcl-2 expression	Burow et al., 2001	V
Suppress the	Human metastatic	Reduced the constitutive	Kanda et al., 2001	V

growth of melanoma	melanoma	interleukin-8 secretion and mRNA levels		
Unrecordered	Mouse IC-21 macrophages	Increased intracellular free Ca2+ concentration ([Ca2+]i)	Peter et al., 2001	i, iv, v
Increase in the frequency of glucose-induced [Ca2+]i oscillations in insulin-releasing	ß-cells within the pancreatic islet of Langerhans	Increased intracellular calcium concentration ([Ca2+]i)	Ropero et al., 2002	i, iv, v
Stimulation of cAMP production	Human SKBR3 breast cancer cells	Via GPR30 to stimulate adenylate cyclase	Filardo et al., 2002	i, v
Formation of membrane ruffles and pseudopodia	MCF-7 cells	Shc phosphorylation, presumably by a Src protein family member, phosphorylation of ERK-1/-2	Song et al., 2002	i, v
E2-induced ERbeta up-regulation	DLD-1 colon cancer cells	E2-induced persistent and palmitoylation-dependent p38/MAPK activation	Caiazza et al., 2007	i
Improves macrophages cytokine production	Splenic macrophages of Male Sprague-Dawley rats	MAPK pathways	Suzuki et al., 2008	iv

Inhibition of colonic contractility	Circular smooth muscle strips of human colon.	Mechanism involving cell membrane coupling	Hogan et al., 2009	i, ii, iv
Inhibits osteoclastic differentiation and the interaction of estrogen receptor-alpha with BCAR1 and Traf6	Human monocytes	RANKL-stimulated	Robinson et al., 2009	I
Induces an antimitogenic effect	Lactotroph cells	Estradiol interacts with insulin through membrane receptors	Gutierrez et al.,	Iv
